# Indoor rock climbing (bouldering) as a new treatment for depression: study design of a waitlist-controlled randomized group pilot study and the first results

**DOI:** 10.1186/s12888-015-0585-8

**Published:** 2015-08-25

**Authors:** Katharina Luttenberger, Eva-Maria Stelzer, Stefan Först, Matthias Schopper, Johannes Kornhuber, Stephanie Book

**Affiliations:** Department of Medical Psychology and Medical Sociology, Friedrich-Alexander-Universität Erlangen-Nürnberg, Clinic for Psychiatry and Psychotherapy, Schwabachanlage 6, 91054 Erlangen, Germany

## Abstract

**Background:**

Depression is one of the most common diseases in industrialised nations. Physical activity is regarded as an important part of therapeutic intervention. Rock climbing or bouldering (rock climbing to moderate heights without rope) comprises many aspects that are considered useful, but until now, there has been hardly any research on the effects of a bouldering group intervention on people with depression. The purpose of this controlled pilot study was twofold: first, to develop a manual for an eight-week interventional program that integrates psychotherapeutic interventions in a bouldering group setting and second, to assess the effects of a bouldering intervention on people with depression.

**Methods:**

The intervention took place once a week for three hours across a period of eight weeks. Participants were randomly assigned to the two groups (intervention vs. waitlist). The intervention group began the bouldering therapy immediately after a baseline measurement was taken; the waitlist participants began after an eight-week period of treatment as usual. On four measurement dates at eight-week intervals, participants completed the Beck Depression Inventory II (BDI-II), the symptom checklist-90-R (SCL-90), the questionnaire on resources and self-management skills (FERUS), and the attention test d2-R. A total of 47 participants completed the study, and the data were analysed with descriptive statistics. Cohen’s d was calculated as a measure of the effect size. For the primary hypothesis, a regression analysis and the Number Needed to Treat (NNT) (improvement of at least 6 points on the BDI-II) were calculated.

**Results:**

After eight weeks of intervention, results indicated positive effects on the measures of depression (primary hypothesis: BDI-II: Cohen’s d = 0.77), this was supported by the regression analysis with “group” as the only significant predictor of a change in depression (*p* = .007). The NNT was four.

**Conclusions:**

These findings provide the first evidence that therapeutic bouldering may offer an effective treatment for depression. Further research is required.

**Trial registration:**

Current controlled trials, ISRCTN17623318, registered on July 15^th^ 2015.

## Background

Depression is one of the most common diseases worldwide with a one-year prevalence of 3.2 % according to the WHO World Health Survey 2007 [1]. It is one of the chronic illnesses that causes the greatest decrement in health [1]. In recent decades, there has been growing evidence [2–10] that physical activity has an important influence on mood, and thus, it has been proposed as a potential treatment for depression [11]. Various studies have shown that under certain circumstances, the effect sizes for physical activities are in the same range as for antidepressants [2, 4, 5, 10] or psychotherapy [4, 5, 10]. Most studies have analysed aerobics or walking [4]. Physical activity seems to be more effective if it is conducted in groups (higher endorphin release [2, 12]) and if it is done regularly [13, 14]. Exercises that require coordination seem to have specific effects on cognitive abilities [15] such as concentration. Furthermore, greater improvements have been found for supervised exercise training as compared with home-based exercise [16], for activity programs that are tailored to specific individuals/groups vs. more generic interventions [17, 18], as well as for manualised psycho-educational interventions compared with interventions that are not accompanied by psychosocial support [19].

Rock climbing or bouldering combines many of these aspects because rock climbing requires high concentration, can be varied according to the fitness level of the person, needs a high level of coordination, can easily be carried out in groups, and activates intense emotions (such as fear, pride, lust, anger, and more). With the expansion of bouldering as a sport for everybody, it seems a logical development to use the positive aspects of bouldering as a therapy for mental illnesses. While some psychiatric hospitals in Germany already use rock climbing as a therapeutic approach, to date, there have been only case reports or small observational studies on the effects of bouldering or rock climbing in the psychotherapeutic field [20–23]. These studies on therapeutic climbing suggest that there might be positive effects on anxiety [20], ADHS [23], depression [20–23], cognition [22], self-esteem [20, 21], as well as in the social domain [22].

Hence, the purpose of this controlled pilot study was first to develop a manual for an 8-week interventional bouldering program for people with depression in an outpatient setting and second to assess the effects of this bouldering intervention on people with depression.

## Methods

### Bouldering intervention: therapy manual

Bouldering is defined as rock climbing to moderate heights (up to around four metres) without rope. Boulder Gyms offer a great number of routes that vary in difficulty levels (often marked by different colours). People of different fitness levels can therefore easily boulder together in the same group without being underchallenged or overstrained. Our newly developed bouldering therapy consisted of eight consecutive weekly sessions of three hours each from ten a.m. to one p.m. An average of 12 to 13 people attended each therapy group at a time, and each session was supervised by two therapists. Each session began with a short meditation or mindfulness exercise; thereafter, the subject of the specific session was given, followed by a short psychoeducation on this subject (for example: How to cope with anxiety). The session proceeded with subject-related bouldering games or exercises. Participants were encouraged to engage in new experiences (for example, bouldering blindfolded). After a break, the last part of the session consisted of free bouldering by which participants in small groups worked on their individual projects supported by the therapists. Each session ended with another mindfulness meditation and a gathering about what was experienced and how this could be integrated into daily life. Table 1 provides an overview of the sessions and their topics. The therapists were mental health therapists (psychologists or registered nurses with a specific psychiatric qualification) who had undergone training in “Therapeutic rock climbing” at the Austrian “Institute for Therapeutic rock climbing” (www.therapieklettern.com). One of the therapists in each session was a climbing instructor certified by the German Alpine Association. The therapists also had several years of climbing experience themselves.

**Table 1:** Session overview and subjects

Session	Topic
1	Introduction to bouldering, support for group cohesion, obtaining an overview of the physical abilities of the participants
	● Introduction to mindfulness-breathing techniques
	● First steps into bouldering: safety rules, getting to know the place, spotting, difficulty of routes
	● First experiences with bouldering, sharing
2	Old habits – new ways
	● Body perception in shifting the focus
	● Bouldering techniques II: Self-awareness, body perception, centre of gravity. Focussing on legs instead of arms
	● Different ways of bouldering the same boulder: old habits vs. new possibilities
3	Expectation versus experience, healthy handling of limitations
	● Focussing on the moment: what are my expectations of me?
	● Feelings of limitation: when is it better to push, when to ease up?
	● Bouldering techniques III: different possibilities for holding and stepping
4	Self-efficacy: the power of small steps
	● Self-efficacy and one’s own experiences
	● Bouldering techniques IV: twisting and Egyptian
5	Fear and trust
	● Fear, anxiety, and panic: what to do?
	● Breathing and other techniques when experiencing fear
	● Differences between objective risks and false alarms
6	Trusting yourself and trusting others
	● Acknowledging and accepting your own limits
	● Accepting help from others
	● Handling the emotions of shame or disappointment
7	Transfer to daily life
	● Sharing of lessons learned
	● One’s own daily life problems: transferring to bouldering situations and back?
8	Reflection of lessons learned, free topic (reflecting the group’s wishes)

### Methods of evaluation

#### Design

**Fig. 1:**
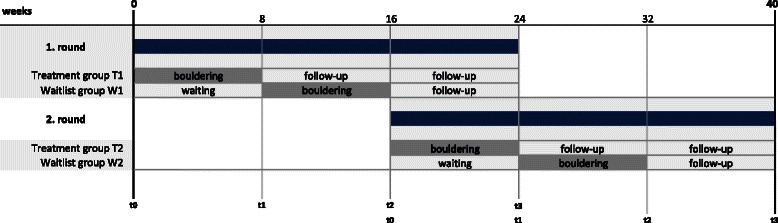
Study design

The study was conducted as a randomised waitlist-controlled pilot study with an intervention period of 8 weeks. After the initial assessment, the intervention group began with the bouldering therapy. After 8 weeks, the groups changed, and the intervention was provided to the waitlist group, while the intervention group returned to their individual treatments that were not influenced by the study. The measurement points consisted of a baseline measure (t0) and measures taken after 8 weeks (t1), 16 weeks (t2), and 24 weeks (t3). See Fig. 1 for the study design. The ethical committee of the Friedrich-Alexander University Erlangen-Nuremberg approved the study design (Re.-No. 99_13 B).

#### Recruitment and randomisation

**Fig. 2:**
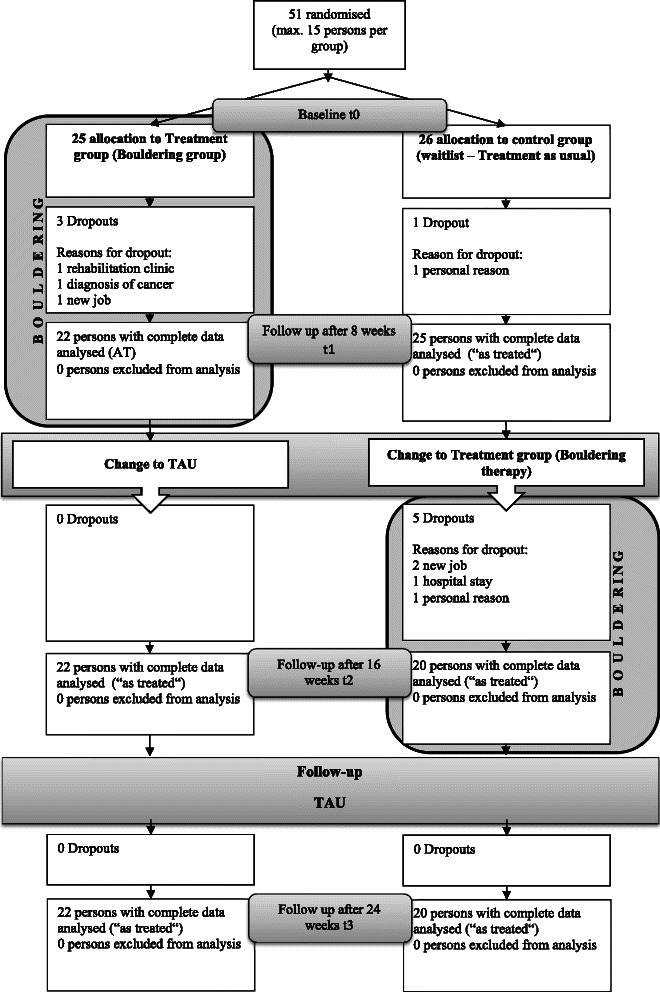
Consort flow chart

Participants were recruited for the study in four different ways: In the two psychiatric hospitals in Erlangen, informational material was laid out and contact details were given so that either the participants or their physicians could apply for study participation. In addition, the same informational material was sent to all psychotherapists in town and also to other services that provide care for people with depression, such as self-help groups or other initiatives. Furthermore, nonbinding informational events were announced in newspapers and via the Internet so that any interested people could participate. Interested participants were informed about the study by the therapists in a face-to-face meeting and provided written consent. They were randomly assigned to one of the two groups: intervention or waitlist. We computer generated a randomisation list for each group at t0 (Treatment group1 (T1) and Waitlist group 1 (W1) together and groups T2 and W2 together; see Consort Flow Chart Fig. 2), assigning half of the participants to the treatment group and the other half to the waitlist group. In some cases, randomisation was not possible because the maximum number of participants had been reached in a group; in a few cases, if a participant was not available on more than two Thursday mornings in one of the two eight-week periods, he or she was assigned to the other time period. Baseline data were collected from all participants, including the WHO screening test on depression (WHO-5 www.who-5.org).

#### Inclusion and exclusion criteria

The inclusion criteria consisted of either a diagnosis of depression by a psychiatrist or less than 13 points on the WHO depression scale [24], informed consent, and having free time on Thursday mornings during the intervention period. Exclusion criteria consisted of undergoing in-patient treatment during either the intervention or the waiting periods, acute suicidality or psychosis, or a strong medical contraindication against sport, determined by a GP or psychiatrist.

#### Instruments

In the beginning, participants completed a questionnaire to collect the following information: age, gender, educational level, employment status, current medication, current psychotherapy, BMI, and experience with rock climbing or bouldering. The participants were also asked to use a 4-point scale (ranging from agree completely to disagree completely) to rate whether they had respect for bouldering and if they slept well at night. In addition, the WHO questionnaire on well-being (www.who-5.org), a short screening tool for depression, was administered to determine participants’ current level of depression and subsequent inclusion in the program.

The BDI-II [25, 26] is a widely used instrument that is designed to measure the intensity of depression experienced during the past two weeks. The BDI-II contains 21 specific symptoms of depression with answer options that consist of four increasing levels of severity, ranging from zero to three. The total score is the sum of all responses, which can range from zero to 63. Scores ranging from zero to 13 indicate minimal depressive symptoms, scores of 14 to 19 represent mild depression, scores of 20 to 28 indicate moderate depression, and scores of 29 or above represent severe depression.

The Symptom-Checklist SCL-90-R [27, 28] is a self-report inventory that is used to examine the global intensity of psychological symptoms and distress experienced during the past seven days using a five-point Likert-type scale ranging from zero to four. The SCL-90-R covers nine symptom dimensions, including depression and anxiety. Ratings are summed for each subscale with higher scores indicating an increasing severity of symptoms.

The FERUS is a widely applied instrument designed to measure individuals’ health-related resources and manageability [29]. Its 66 items comprise seven scales, including subscales that measure self-efficacy, coping, and self-verbalisation. Items are rated on a 5-point Likert scale that ranges from one to five, with higher test scores indicating better resources and manageability skills.

The d2-R, a paper-pencil test consisting of 14 lines with 57 characters each, was administered to measure participants’ attention and concentration performance [30]. Individuals were instructed to discriminate between similar visual stimuli by crossing out target objects (d with two lines) while ignoring other characters (p or d with no, less than two, or more than two lines). Scores provided by the d2-R include concentration performance (CP), percentage of errors (E %), fluctuation rate (FR), as well as the total number of items processed minus errors (TN-E).

#### Statistical analyses

The analyses were carried out with SPSS 21.0. Descriptive methods were used for the sample description and the presentation of the results (frequencies, percentages, means, and standard deviations). First, we computed difference scores as the difference between t1 and t0. These difference scores (i.e. change scores over the intervention period for the intervention group versus over the waiting period for the waitlist group) were compared with a two-sample T-Test (after checking for homogeneity of variance). As a sensitivity analysis, U-Tests were also computed. Cohen’s d was calculated as a measure of effect size. For the main outcome criterion (depression measured with the BDI-II), a regression analysis was computed with age, sex, medication (antidepressants yes or no), psychotherapy, depression severity, and group as predictors. In addition, the Number Needed To Treat was calculated. An improvement in the BDI-II of more than 6 points was defined as a clinically relevant threshold. This reflects an improvement of about one severity grade. Secondary outcomes were viewed as exploratory.

## Results

### Sample

The sample was comprised of four groups, i.e. two intervention and two waitlist groups, 51 participants altogether, who completed their study period between July 2013 and May 2014. In data analysis the 2 intervention groups and the 2 waitlist groups are taken together. During the first 16 weeks, 9 participants dropped out, three in the intervention group and 6 in the waitlist group. Their reasons for dropping out during the intervention were mainly due to conflicts in schedules, as three participants were able to return to their jobs and another two were placed in rehabilitation centres. They were no longer able to attend the bouldering intervention, which was held from 10 a.m. to 1 p.m. One participant had to stay home because she had just been diagnosed with cancer and another because she had to care for her husband who became terminally ill. Another two quit for personal reasons. (See Fig. 2 for the CONSORT Flow Chart).

Of the 47 participants remaining after the first 8 weeks, 27 were female and 20 were male with an average age of 44 years and an average WHO score of 8.45. Half of the participants underwent psychotherapeutic treatment in addition to their study participation, and almost 70% took antidepressive medication. Only 2 participants – one in each group – received neither psychotherapy nor medical treatment. Neither the psychotherapeutic nor the pharmacological treatments were influenced by the study. See Table 2 for the sample description. All analyses that included only the data from the t0 and t1 timepoints were computed on these 47 participants.

**Table 2:** Sample characterisitics (*n* = 47)

Variable	Intervention group		Waitlist group		Total		Test of group differences		
	(*n* = 22)		(*n* = 25)		(*n* = 47)		χ^2^	*U*	*p*
Age^a^, *M* (*SD*)	42.71	(11.88)	44.96	(12.08)	43.91	(11.91)		242.50	.49
Sex, *n* (%)							0.14		.71
Women	12	(54.5)	15	(60.0)	27	(57.5)			
Men	10	(45.5)	10	(40.0)	20	(42.5)			
School education, *n* (%)							3.84		.43
8 years	1	(4.5)	2	(8.0)	3	(6.4)			
10 years	3	(13.6)	7	(28.0)	10	(21.3)			
13 years	3	(13.6)	5	(20.0)	8	(17.0)			
Vocational training	4	(18.2)	5	(20.0)	9	(19.1)			
University	11	(50.0)	6	(24.0)	17	(36.2)			
Additional psychotherapy (*n* (%)							0.47		.49
yes	11	(50.0)	15	(60.0)	26	(55.3)			
no	11	(50.0)	10	(40.0)	21	(44.7)			
Antidepressants, *n* (%)							0.36		.55
yes	15	(68.2)	19	(76.0)	34	(72.3)			
no	7	(31.8)	6	(24.0)	13	(27.7)			
BMI^a^, *M* (*SD*)	26.81	(5.73)	24.56	(3.95)	25.61	(4.94)		201.00	.12
Already some experience with bouldering or rock climbing, *n* (%)							0.10		.75
yes	8	(36.4)	8	(32.0)	16	(34.0)			
no	14	(63.6)	17	(68.0)	31	(66.0)			
WHO well-being scale^a^ *M* (*SD*)	8.86	(4.63)	8.08	(4.93)	8.45	(4.76)		237.50	.42

^a^deviation from normal distribution (Shapiro-Wilk Test)

BMI: Body Mass Index

### Evaluation outcome

There were no differences between the waitlist and intervention groups on the screening or at the first measurement point t0, although the intervention group had a slightly lower BDI sum score than the waitlist (*n* = 47, difference BDI t0: 3.1 points, *p* = .378).

Dropouts (*n* = 9) had the same age (mean 46, median 48 years; U-Test: *p* = .448) and the same severity of depression (BDI-sum mean: 25, median 26; U-Test: *p* = .473). Of the dropouts, 8 were women.

### Main outcome

During the 8-week bouldering therapy, the intervention group’s BDI-II score improved by 6.27 points, but for the same time period, the waitlist group’s BDI-II improved by only 1.4 points, which was significantly less (*n* = 47; T-Test: *p* = .012; U-Test: *p* = .011). The effect size was moderate with a Cohen’s d of .77. In a regression analysis, group allocation was the only significant predictor (*p* = .007) with depression severity showing a trend toward significance *p* = .063 (see Table 3) with those with higher symptom severity scores showing greater improvement compared with those with lower symptom severity. Of the 22 participants in the intervention group, 10 improved by more than one severity grade on the BDI-II (i.e. more than 6 points). Of the 25 participants in the waitlist group, only two did so. This produces a Number Needed to Treat of 4.

**Table 3:** Regression analysis with BDI-II at t1 as the dependent variable

			95 % CI	
Independent variables	Unstand. *b*	*p*	Lower	Upper limit
Sex (female)	−0.76	.705	−4.76	3.25
Age	−0.04	.598	−0.21	0.12
Group allocation (intervention)	−5.39	.007**	−9.24	−1.54
Antidepressive medication	−0.44	.838	−4.72	3.84
Additional psychotherapy	1.41	.484	−2.63	5.46
BDI-II baseline	−0.16	*.063*	−0.32	0.01

**Significant p-values (<.01) are bolded and marked with **. p-values below .1 are italicized

**Fig. 3:**
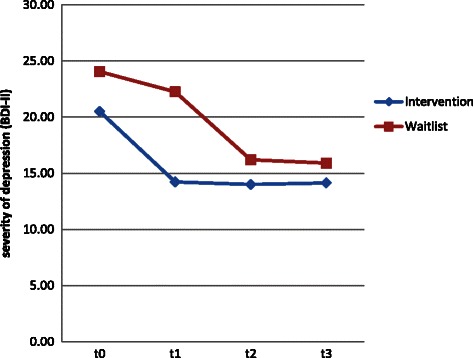
Severity of depression, operationalised by the BDI-II score for 4 measurement points for the intervention (*n* = 22) and waitlist groups (*n* = 20)

During their intervention period (between t1 and t2), the former waitlist group also improved by 6.0 points, whereas the former intervention group remained unchanged after their intervention from t1 to t3 (T-test for dependent samples: Intervention group t3-t1, *p* = .956). The waitlist group also remained unchanged after their intervention for at least 8 weeks (T-test for dependent samples: t3-t2 *p* = .695). The improvement of the former waitlist group was not due to the dropouts because the scores at t1 did not differ between the 25 (with dropouts: BDI-II: 22.2) and 20 persons (without dropouts: BDI-II: 22.3). Figure 3 presents an overview of the course of the BDI-II scores across the 4 measurement points.

### Secondary outcomes

Significant differences between the two groups’ changes after the first 8 weeks were also found for the SCL-subscales “depression” (T-Test: *p* = .041, U-Test: *p* = .036) and “obsessive-compulsive behaviour” (T-Test: *p* = .019, U-Test: *p* = .031) and the FERUS-subscales “self-efficacy” (T-Test: *p* = .037, U-Test: .025) and “active and passive coping” (T-Test: *p* = .010, U-Test: *p* = .017). For an overview of all scales, see Table 4.

**Table 4:** Exploratory outcomes

	Intervention group (*n* = 22)	Waitlist group (*n* = 25)	T-Test for independent samples		*U*-Test		Cohen’s *d*
Scale	*∆M* (*SD*)	*∆M* (*SD*)	*t*	*p*	*U*, z	*p*	
Depression							
BDI-II (primary hypothesis)	−6.27(5.64)	−1.40 (6.94)	*t*(45) = 2.62	.012*	*U* = 156.50, z = -2.53	.011*	0.77
Depression (SCL-90-R)	−4.55 (4.38)	−1.96 (4.06)	*t*(45) = 2.10	.041*	*U* = 177.00, z = -2.10	.036*	0.62
Anxiety							
Phobic anxiety (SCL-90-R)	−2.77 (6.03)	−1.52 (9.13)	*t*(45) = 0.55	.587	*U* = 251.00, z = -0.52	.606	0.16
Anxiety^a^ (SCL-90-R)	−5.09 (8.23)	−1.28 (6.48)	*t*(45) = 1.77	.083	*U* = 182.50, z = -1.98	.048*	0.52
Social competence							
Interpersonal sensitivity (SCL-90-R)	−5.23 (7.07)	−1.76 (4.63)	*t*(45) = 2.01	.050	*U* = 191.00, z = -1.80	.071	0.59
Social support^a^ (FERUS)	0.95 (8.85)	2.20 (5.13)	*t*(45) = 0.60	.552	*U* = 236.50, z = -0.83	.409	0.18
Self-management							
Active and passive coping (FERUS)	6.32 (8.49)	0.60 (5.39)	*t*(34.74) = -2.71	.010*	*U* = 163.50, z = -2.39	.017*	0.82
Self-efficacy (FERUS)	3.72 (6.96)	−0.20 (5.58)	*t*(45) = -2.15	.037*	*U* = 171.50, z = -1.24	.025*	0.63
Self-verbalisation (FERUS)	3.91 (6.16)	0.80 (6.06)	*t*(45) = -1.74	.089	*U* = 195.50, z = -1.71	.088	0.51
Concentration							
Concentration^a^ (d2-R)	2.09 (6.02)	1.12 (10.01)	*t*(45) = -0.40	.694	*U* = 271.00, z = -0.09	.932	0.12
Further outcomes							
Obsessive-compulsive^a^ (SCL-90-R)	−4.77 (4.80)	−1.80 (3.25)	*t*(36.23) = 2.45	.019*	*U* = 175.00, z = -2.16	.031*	0.73

Comparison of differences between t0 and t1. Negative values on the SCL-90-R indicate improvements in symptoms, positive values on the FERUS and d2 indicate improvements in abilities

^a^deviations from a normal distribution (Shapiro-Wilk Test)

*p-values < .05

## Discussion

To our knowledge, this is the first article to address a bouldering or rock climbing group therapy for people with depression using a controlled design. We found that depressive symptoms given by self-report can be reduced on average by 6 points on the BDI-II by applying an 8-week bouldering psychotherapy program in groups of 10 to 14 participants. The effect sizes (Cohen’s d) in this pilot study were comparable to other short-term group therapies [31] and to meta-analyses that have reported effect sizes for physical activity in depression of 0.53 to 1.1 [9, 32–34]. In contrast to the public’s impression (likely evoked by reports on free solo climbing or extreme climbing), indoor bouldering is a comparatively safe sport, and the most common injuries concern bruises. The easiest routes in indoor bouldering gyms can be mastered even by most untrained participants. While every bouldering gym is equipped with large mattresses to soften possible jump-downs, when choosing a gym for bouldering therapy, therapists should choose an indoor gym that offers the opportunity for participants to step out of the route and come down via a ladder or stairs.

A current study on the incidence of climbing-associated injuries found that the average was 0.2 injuries per 1,000 h of outdoor rock climbing [35]. In a supervised indoor bouldering setting, this should be even less, but of course all participants must adhere to the safety rules given by the therapists, and therapists must be trained in climbing safety.

In recent years, there has already been some interest in the therapeutic use of rock climbing as a treatment for depression, reflected by a number of case reports and theoretical discussions in journals [22, 23] and on the Internet. Nevertheless, we found only two published studies in which participants’ data were analysed before and after the rock climbing therapy [20, 21]. Only one used standardised questionnaires [20] but did so in a direct pre-post design after one session of a tightrope course. The control group was comprised of participants who were not able or did not want to participate in the course. Other reports used self-developed questionnaires and had no control group. We did not find any study that employed a follow-up.

In our study, for the first 8 weeks, the intervention group underwent the bouldering therapy, which was conducted at a local bouldering gym and consisted of 8 sessions of three hours each. For that time period, the control group were administered their treatment as given by their individual psychiatrist or psychotherapist. After eight weeks, the groups changed. The intervention group was followed up 8 weeks after the end of therapy, and again, 16 weeks after the end of therapy. Both groups improved during their intervention period with a significant difference between the intervention and the waitlist group during the first 8 weeks, thus providing support for the effectiveness of a standardised bouldering therapy for people with depression. To date, little is known about the underlying mode of action of the effectiveness of physical activity in treating depression. Why might a bouldering therapy be effective? Certainly the physical activity itself has a positive influence on the depressive symptoms as already shown in different reviews [4, 9]. In contrast to most studies in which exercise interventions consisted of running or aerobics, aimed at improving or maintaining one or more components of physical fitness [4], bouldering focusses in particular on mental aspects. For this reason, bouldering may be especially interesting as a therapeutic tool since many people with depression have poor physical health, low levels of fitness and physical self-worth, and less motivation for heavy physical effort [10]. Moreover, patients with depression accumulate a lot of barriers for participation in exercise interventions (e.g. psychosomatic complaints, low self-confidence) [10]. Thus, it is strongly recommended that they have a conversation about barriers and possible strategies [10] as such conversations are a permanent component of our suggested bouldering therapy. In addition, we hypothesise that bouldering enhances feelings of self-efficacy as the mastering of “bouldering problems” can be seen and felt directly and within a short amount of time. Our data suggest that this hypothesis might be correct because, after the intervention, the bouldering group had a significantly higher feeling of self-efficacy than the waitlist group. As in all group therapy, there is a great influence of social contact with other participants. This is especially encouraged in bouldering therapy as participants are trained to support each other, to work together on bouldering problems, and to provide feedback and applause. Social interaction might therefore be a strong therapeutic component of the bouldering therapy. This idea is supported by the data, which showed a significant increase in coping strategies and a trend toward diminished interpersonal sensitivity. Another hypothesis focusses on the mindfulness that is necessary while bouldering and that is stimulated by the meditation exercises. Given that one of the main symptoms of depression is rumination, strengthening mindfulness and concentration has often been shown to be an efficient therapeutic approach [36–38]. In contrast to other sports (e.g. running or cycling), bouldering challenges not only the physical but also the cognitive and emotional resources of the individual. This hypothesis should be tested in future studies.

### Strengths and limitations

One strength of the study is the controlled and randomised design and the relatively long follow-up period of 8 to 16 weeks. Limitations consist of using the control group as a waitlist group, the small sample size, and the assessment of symptoms via only self-report.

The waitlist group began with a somewhat higher BDI score, which could have influenced the outcome. On the other hand, the control group improved during their own intervention period as much as the intervention group did, and the results of the regression analysis showed a trend such that those with higher symptom severity scores showed greater improvement than those with lower symptom severity. Therefore, the baseline differences did not seem to have influenced the results in favor of the intervention. Recruiting participants via the clinical outpatient centre played a larger role than it would have in a non-hospital setting. Hence, the current sample might be different from participants without hospital experience.

We excluded patients with acute suicidality, psychosis, or a strong medical contraindication against sport as determined by their GP or psychiatrist. Future studies should analyse the responses of different patient groups to the bouldering therapy according to different medical conditions. Therefore, the results of this study also need to be replicated with other participants, and the therapy should be evaluated with respect to its cost-effectiveness. Future researchers should be aware that the therapy described in this study exceeds standard bouldering lessons and that therapists need not only rock climbing or bouldering experience but also a profound psychotherapeutic background. Future studies should additionally compare the bouldering intervention with psychotherapeutic interventions alone or other physical activities and should focus on modes of action.

## Conclusions

This is the first randomised controlled study on a bouldering intervention for people with depression. The short-term bouldering intervention was effective with an effect size of d = .77 in the treatment of depression. Future research is required.

## References

[1] Moussavi S, Chatterji S, Verdes E, Tandon A, Patel V, Ustun B (2007). Depression, chronic diseases, and decrements in health: results from the World Health Surveys. Lancet.

[2] Blumenthal JA, Babyak MA, Doraiswamy PM, Watkins L, Hoffman BM, Barbour KA, Herman S, Craighead WE, Brosse AL, Waugh R (2007). Exercise and pharmacotherapy in the treatment of major depressive disorder. Psychosom Med.

[3] Silveira H, Moraes H, Oliveira N, Coutinho ES, Laks J, Deslandes A (2013). Physical exercise and clinically depressed patients: A systematic review and meta-analysis. Neuropsychobiology.

[4] Cooney GM, Dwan K, Greig CA, Lawlor DA, Rimer J, Waugh FR, McMurdo M, Mead GE (2013). Exercise for depression. Cochrane Database Syst Rev.

[5] Rimer J, Dwan K, Lawlor DA, Greig CA, McMurdo M, Morley W, Mead GE (2012). Exercise for depression. Cochrane Database Syst Rev.

[6] Broocks A, Bandelow B, Pekrun G, George A, Meyer T, Bartmann U, Hillmer-Vogel U, Rüther E (1998). Comparison of aerobic exercise, Clomipramine, and Placebo in the treatment of panic disorder. Am J Psychiatry.

[7] Broocks A (2005). Körperliches Training in der Behandlung psychischer Erkrankungen [Psychological effects of regular exercises]. Bundesgesundheitsbl - Gesundheitsforsch -Gesundheitsschutz.

[8] Blake H (2012). Physical activity and exercise in the treatment of depression. Front Psychiatry.

[9] Wegner M, Helmich I, Machado S, Nardi AE, Arias-Carrion O, Budde H (2014). Effects of exercise on anxiety and depression disorders: Review of meta-analyses and neurobiological mechanisms. CNS Neurol Disord Drug Targets.

[10] Knapen J, Vancampfort D, Morien Y, Marchal Y. Exercise therapy improves both mental and physical health in patients with major depression. Disabil Rehabil. 2014;1–6. 10.3109/09638288.2014.972579 25342564

[11] Trivedi MH, Greer TL, Grannemann BD, Chambliss HO, Jordan AN (2006). Exercise as an augmentation strategy for treatment of major depression. J Psychiatr Pract.

[12] Cohen EEA, Ejsmond-Frey R, Knight N, Dunbar RIM (2010). Rowers’ high: Behavioural synchrony is correlated with elevated pain thresholds. Biol Lett.

[13] Ernst C, Olson AK, Pinel JP, Lam RW, Christie BR (2006). Antidepressant effects of exercise: evidence for an adult-neurogenesis hypothesis?. J Psychiatry Neurosci.

[14] Perraton LG, Kumar S, Machotka Z (2010). Exercise parameters in the treatment of clinical depression: a systematic review of randomized controlled trials. J Eval Clin Pract.

[15] Voelcker-Rehage C, Godde B, Staudinger UM (2011). Cardiovascular and coordination training differentially improve cognitive performance and neural processing in older adults. Front Hum Neurosci.

[16] Kodis J, Smith KM, Arthur HM, Daniels C, Suskin N, McKelvie RS (2001). Changes in exercise capacity and lipids after clinic versus home-based aerobic training in coronary artery bypass graft surgery patients. J Cardiopulm Rehabil.

[17] Segar M, Jayaratne T, Hanlon J, Richardson CR (2002). Fitting fitness into women’s lives: effects of a gender-tailored physical activity intervention. Womens Health Issues.

[18] Strecher V, Wang C, Derry H, Wildenhaus K, Johnson C (2002). Tailored interventions for multiple risk behaviors. Health Educ Res.

[19] Callaghan P, Khalil E, Morres I, Carter T (2011). Pragmatic randomised controlled trial of preferred intensity exercise in women living with depression. BMC Public Health.

[20] Mehl K, Wolf M (2008). Erfahrungsorientiertes Lernen in der Psychotherapie - Evaluation psychophysischer Expositionen auf dem Hochseil im Rahmen eines multimethodalen stationären Behandlungskonzeptes [Experiential learning in psychotherapy. Evaluation of psychophysical exposure to a tightrope course as adjunct to inpatient psychotherapeutic treatment]. Psychotherapeut.

[21] Mollenhauer A, Doll N, Renz P, Luntz J (2011). Therapeutisches Klettern in der Akutpsychiatrie [Therapeutic climbing for acute psychiatric patients]. Pflegewissenschaft.

[22] Schnitzler EE (2009). Loslassen, um weiter zu kommen - Praxisbericht: Therapeutisches Klettern in der psychosomatischen Rehabilitation [Letting go in order to move on--clinical report: therapeutic climbing in psychosomatic rehabilitation]. Rehabilitation.

[23] Wallner S. Psychologisches Klettern: Klettern als Mittel klinisch- und gesundheitspsychologischen Handelns [Psychological Climbing. Climbing as an instrument of clinical and health psychological treatment]. Psychologie in Österreich. 2010;30(5):396-403

[24] Bech P (2004). Measuring the dimensions of psychological general well-being by the WHO-5. Qual Life Newsl.

[25] Hautzinger M, Keller F, Kühner C (2006). Beck Depressions-Inventar (BDI-II). Revision.

[26] Beck AT, Steer RA, Brown G (1996). Manual for the Beck Depression Inventory-II.

[27] Franke GH (2002). Die Symptom-Checkliste von L. R. Derogatis (2. vollständig überarbeitete und neu normierte Auflage).

[28] Schmitz N, Hartkamp N, Kruse J, Franke GH, Reister G, Tress W (2000). The Symptom-Check-List-90-R (SCL-90-R): A German validation study. Qual Life Res.

[29] Jack M (2007). Fragebogen zur Erfassung von Ressourcen und Selbstmanagementfähigkeiten (FERUS).

[30] Brickenkamp R, Schmidt-Atzert L, Liepmann D (2010). d2-R Test - Revision (Aufmerksamkeits- und Konzentrationstest) [Attention- and Concentration Test d2 - revised version].

[31] McDermut W, Miller IW, Brown RA (2001). The efficacy of group psychotherapy for depression: A meta-analysis and review of empirical research. Clin Psychol Sci Pract.

[32] Lawlor DA, Hopker SW (2001). The effectiveness of exercise as an intervention in the management of depression: systematic review and meta-regression analysis of randomised controlled trials. BMJ.

[33] Josefsson T, Lindwall M, Archer T (2014). Physical exercise intervention in depressive disorders: meta-analysis and systematic review. Scand J Med Sci Sports.

[34] North TC, McCullagh P, Tran ZV (1990). Effect of exercise on depression. Exerc Sport Sci Rev.

[35] Neuhof A, Hennig FF, Schoffl I, Schoffl V (2011). Injury risk evaluation in sport climbing. Int J Sports Med.

[36] Kenny MA, Williams JM (2007). Treatment-resistant depressed patients show a good response to Mindfulness-based Cognitive Therapy. Behav Res Ther.

[37] Hofmann SG, Sawyer AT, Witt AA, Oh D (2010). The effect of mindfulness-based therapy on anxiety and depression: A meta-analytic review. JCCP.

[38] Baer RA. Mindfulness training as a clinical intervention: A conceptual and empirical review. Clin Psychol Sci Pract. 2003, 10(2):125–43.

